# Bioprocess development for universal influenza vaccines based on inactivated split chimeric and mosaic hemagglutinin viruses

**DOI:** 10.3389/fbioe.2023.1097349

**Published:** 2023-06-05

**Authors:** Eduard Puente-Massaguer, Annika Beyer, Madhumathi Loganathan, Iden Sapse, Juan Manuel Carreño, Goran Bajic, Weina Sun, Peter Palese, Florian Krammer

**Affiliations:** ^1^ Department of Microbiology, Icahn School of Medicine at Mount Sinai, New York, NY, United States; ^2^ Center for Vaccine Research and Pandemic Preparedness (C-VaRPP), Icahn School of Medicine at Mount Sinai, New York, NY, United States; ^3^ Department of Medicine, Icahn School of Medicine at Mount Sinai, New York, NY, United States; ^4^ Department of Pathology, Molecular and Cell-Based Medicine, Icahn School of Medicine at Mount Sinai, New York, NY, United States

**Keywords:** bioprocess, HA stalk, neuraminidase, NA activity, cHA, mHA, beta-propiolactone, Triton X-100

## Abstract

Seasonal influenza viruses account for 1 billion infections worldwide every year, including 3–5 million cases of severe illness and up to 650,000 deaths. The effectiveness of current influenza virus vaccines is variable and relies on the immunodominant hemagglutinin (HA) and to a lesser extent on the neuraminidase (NA), the viral surface glycoproteins. Efficient vaccines that refocus the immune response to conserved epitopes on the HA are needed to tackle infections by influenza virus variants. Sequential vaccination with chimeric HA (cHA) and mosaic HA (mHA) constructs has proven to induce immune responses to the HA stalk domain and conserved epitopes on the HA head. In this study, we developed a bioprocess to manufacture cHA and mHA inactivated split vaccines and a method to quantify HA with a prefusion stalk based on a sandwich enzyme-linked immunosorbent assay. Virus inactivation with beta-propiolactone (βPL) and splitting with Triton X-100 yielded the highest amount of prefusion HA and enzymatically active NA. In addition, the quantity of residual Triton X-100 and ovalbumin (OVA) was reduced to very low levels in the final vaccine preparations. The bioprocess shown here provides the basis to manufacture inactivated split cHA and mHA vaccines for pre-clinical research and future clinical trials in humans, and can also be applied to produce vaccines based on other influenza viruses.

## 1 Introduction

Current seasonal influenza virus vaccines are effective when they are well matched to circulating strains (Tricco et al., 2013). The immunity induced by these vaccines is mainly focused on eliciting neutralizing antibodies against the head domain of the surface glycoprotein hemagglutinin (HA) (Becker et al., 2021). The head domain of the HA is highly plastic (Heaton et al., 2013; Kirkpatrick et al., 2018) and tolerates mutations that help the virus to escape from pre-existing immunity, a process known as antigenic drift. This leads to strain specific responses and current vaccines are only effective when well matched with circulating strains. Vaccine effectiveness drops sharply against drift variants or new pandemic influenza viruses. In contrast, the HA stalk domain is relatively conserved within each influenza virus group (group 1: H1, H2, H5, H6, H8, H9, H11, H12, H13, H16, H17, H18, group 2: H3, H4, H7, H10, H14, H15), but immunosubdominant (Krammer and Palese, 2019). Several strategies have been developed to overcome the limitations of current commercial vaccines by refocusing the immune response toward the HA stalk domain. One of these approaches is based on sequential vaccination with chimeric HA (cHA) constructs in which the HA head domain of seasonal influenza viruses has been replaced by head domains belonging to influenza virus subtypes that do not circulate in humans (Hai et al., 2012). A similar strategy based on mosaic HA (mHA) has been developed for influenza B viruses in which the immunodominant epitopes in the head domain of the HA have been replaced by the corresponding HA epitopes from influenza virus subtypes to which humans are naïve (Sun et al., 2019). Vaccination with these constructs provides protection in different animal models (Krammer et al., 2013; Krammer et al., 2014; Ryder et al., 2015; Nachbagauer et al., 2016; Ermler et al., 2017; Nachbagauer et al., 2017; Isakova-Sivak et al., 2018; Nachbagauer et al., 2018; McMahon et al., 2019; Liu et al., 2021a; Liu et al., 2021b; Bliss et al., 2022), and elicits durable and cross-reactive immune responses in clinical trials in humans (Bernstein et al., 2020; Nachbagauer et al., 2021; Folschweiller et al., 2022). The titer of HA stalk antibodies is also an independent correlate of protection from influenza virus infection (Ng et al., 2019).

Inactivated split vaccines have been shown to be an excellent platform to elicit anti HA stalk antibodies in humans (Bernstein et al., 2020; Nachbagauer et al., 2021). Moreover, the production of inactivated split vaccines in embryonated chicken eggs is currently the workhorse manufacturing platform for seasonal influenza vaccines (e.g., Afluria^®^, Fluad^®^, Fluarix^®^, FluLaval^®^, Fluzone^®^) (Grohskopf et al., 2018). By the initial inactivation step, the virus is rendered unable to infect or replicate, while virus splitting entails virus breakage into smaller parts to reduce vaccine reactogenicity (Kon et al., 2016). Formaldehyde (FA) and beta-propiolactone (βPL) are the alkylating agents most frequently used for virus inactivation (Sabbaghi et al., 2019). Detergents such as Triton X-100, and sodium deoxycholate (DCO) are usually employed for virus splitting. In this study, we report the development of a bioprocess to produce inactivated split cHA and mHA vaccines for pre-clinical testing, and to support the transfer of this technology to larger production scales. We tested the impact of virus inactivation with FA and βPL, and splitting with Triton X-100 and DCO on the amount of prefusion HA in group 1 cHA, group 2 cHA, and influenza B mHA viruses. We also characterized the impact of inactivation and splitting on the enzymatically active neuraminidase (NA) and on virus structure and morphology. The level of residual contaminants in the intermediate and final products were also analyzed. Furthermore, we developed a method based on a sandwich enzyme-linked immunosorbent assay to quantify HA with a prefusion stalk domain in our cHA and mHA virus samples.

## 2 Materials and methods

### 2.1 Cells, viruses, recombinant proteins

Sf9 cells (CRL-1771, ATCC) for baculovirus generation and amplification were grown in *Trichoplusia ni* medium-formulation Hink insect cell medium (TNM-FH, Gemini Bioproducts) supplemented with 10% v/v fetal bovine serum (FBS, Sigma-Aldrich), penicillin (100 U/mL) -streptomycin (100 μg/mL) solution (Gibco), and 0.1% v/v Pluronic F-68 (Gibco). High Five cells (BTI-*TN*-5B1-4, B85502, Thermo Fisher Scientific) were used for recombinant hemagglutinin (rHA) production and were grown in Express Five SFM (Gibco) supplemented with 16 mM L-glutamine (Gibco) (Margine et al., 2013a).

Viruses expressing different chimeric hemagglutinin (cHA) proteins were generated by reverse genetics as previously reported (Hai et al., 2012; Margine et al., 2013b; Liu et al., 2021a). For group 1 cHA viruses, the H8 head domain of the cH8/1_Cal09_N1_PR8_ virus was derived from the HA of A/mallard/Sweden/24/2002 H8N4 virus, and the H5 head domain of the cH5/1_Cal09_N1_Cal09_ virus from A/Vietnam/1203/2004 (H5N1) virus. The HA stalk domain of both viruses was derived from A/California/04/2009 (H1N1) virus, with the NA of the cH5/1_Cal09_N1_Cal09_ virus derived from this same virus, and the NA of the cH8/1_Cal09_N1_PR8_ virus derived from the high-yielding donor vaccine strain A/Puerto Rico/8/1934 (H1N1) virus. As for group 2 cHA viruses, the H15 head domain of the cH15/3_HK14_N2_HK14_ virus was derived from the HA of A/wedge-tailed shearwater/Western Australia/2576/1979 (H15N9) virus, and the H4 head domain of the cH4/3_HK14_N2_HK14_ virus from A/duck/Czechoslovakia/1956 (H4N6) virus. The HA stalk domain and NA of both viruses was derived from A/Hong Kong/4801/2014 (H3N2) virus. The internal genes of group 1 and 2 cHA viruses were derived from A/Puerto Rico/8/1934 (H1N1) virus. In the case of mHA influenza B viruses, the major HA head antigenic sites of B/Brisbane/60/2008 were replaced by the putative antigenic sites of A/black-headed gull/Sweden/1/1999 (H13N6) virus, resulting in the mH13/B_Brisbane_ virus. For the mH5/B_Phuket_ virus, the major antigenic sites of B/Phuket/3073/2013 were replaced by those from A/Vietnam/1203/2004 (H5N1) virus. For both mHA influenza B viruses, the NA and internal genes were derived from the mouse-adapted B/Malaysia/2506/2004 virus. The sequences of the HA genes of the vaccine strains have been submitted to Genbank under accession numbers listed in the end of the methods section. Work with live virus was performed at appropriate biosafety level in a biosafety cabinet.

The six cHA and mHA viruses were grown in 10-day-old embryonated chicken eggs (Charles River) for 48 h at 37°C or for 72 h at 33°C, respectively. Eggs were then cooled overnight (O/N) at 4°C before collection of the allantoic fluid. The harvested allantoic fluid was clarified at 2000 ×g for 10 min at 4°C to remove debris, aliquoted and stored at −80°C, and titrated by the plaque assay method on Madin Darby canine kidney (MDCK) cells (Hai et al., 2008).

### 2.2 Monoclonal antibodies

Murine monoclonal antibodies (mAbs) 1A7 (directed against a linear epitope on the H8 head) (Rajendran et al., 2018), 1H4 (directed against a linear epitope on the H5 head) (Rajendran et al., 2018), 1G4 (directed against a linear epitope on the H4 head) (Amanat et al., 2019), 6G8, 3G11, 6F6 (directed against linear epitopes on the H15 head), 5C5 (directed against the influenza B virus HA head) (Asthagiri Arunkumar et al., 2019), 9H10 (directed against the H3 stalk) (Tan et al., 2014), 2G4, 4C10, and 4G12 (directed against the influenza B HA stalk) (Asthagiri Arunkumar et al., 2019) were produced from hybridomas previously generated using a classical hybridoma fusion protocol (Rajendran et al., 2018). Human mAbs CR9114 (directed against the HA stalk), CR8033, CR8059 (directed against the influenza B virus HA head) (Dreyfus et al., 2012), and CR3022 [directed against the severe acute respiratory syndrome coronavirus 2 (SARS-CoV-2) receptor binding domain] (ter Meulen et al., 2006) were produced by transient DNA plasmid transfection of Expi293F cells (A14527, Thermo Fisher Scientific). All mAbs were purified via protein G sepharose (GE Healthcare) affinity chromatography (Jungbauer et al., 1989).

Stalk-reactive mAbs were biotinylated for HA quantification via sandwich enzyme-linked immunosorbent assay (ELISA, see section 2.6). Briefly, mAbs were biotinylated with the EZ-link NHS-PEG4-Biotin kit (Thermo Fisher Scientific) using a 20-fold molar excess of biotin. The mAb-biotin mixture was incubated at room temperature (RT) for 30 min, and unbound biotin was removed by size exclusion chromatography with Zeba Spin desalting columns at 1,500 ×g for 1 min (Thermo Fisher Scientific).

### 2.3 Production of inactivated split influenza viruses

The clarified allantoic fluid was initially concentrated 10-fold by tangential flow filtration using a Vivaflow 200 regenerated cellulose membrane (100 kDa, Sartorius) on ice. 25–30 mL of the concentrated virus was loaded on centrifuge tubes (Seton Scientific) containing 5 mL of 30% w/v sucrose cushion prepared in 1X NTE buffer [1 M NaCl, 100 mM Tris-HCl, 10 mM ethylenediaminetetraacetic acid (EDTA) in water for injection (Gibco)] and with the pH adjusted to 7.4. Loaded centrifuge tubes were ultracentrifuged at 25000 rpm, 4 °C for 2 h, and the pelleted virus was resuspended in phosphate-buffered saline (PBS, pH = 7.4, Gibco). The resuspended virus was inactivated with beta-propiolactone (βPL, Sigma-Aldrich) or methanol-free formaldehyde (FA, Polysciences Inc.) prepared in ice-cold water for injection after pH buffering with 0.01 M disodium hydrogen phosphate (Millipore). βPL activity was stopped by incubation at 37 °C for 2 h, whereas FA was removed by diafiltration with 100 kDa centrifugal filters (Millipore). To assess the efficiency of the inactivation step, the highly concentrated virus samples were diluted 1:1,000 in fresh PBS and injected into eggs, incubated at 37 °C for 48 or 72 h, and a hemagglutination (HA) assay was conducted to detect viral replication. After inactivation, the virus sample was centrifuged at 4000 rpm, 4 °C for 30 min. The virus was split with Triton X-100 (TX-100, Fisher Bioreagents) or sodium deoxycholate (DCO, Sigma-Aldrich), and the detergent was removed by incubation with 0.2–0.3 g of bio-beads SM-2 (BioRad) per mL of inactivated split virus at 4 °C O/N. As for DCO treated viruses, a pre-incubation step of the bio-beads with DCO split viruses at 37 °C was conducted to avoid sample solidification. The supernatant was collected, and the total protein concentration was adjusted to 0.5–1 mg/mL with PBS (pH = 7.4) using the Bradford assay (BioRad). Samples were aliquoted to 50–100 µL and stored at −80 °C until analysis.

### 2.4 Hemagglutination assay

The presence of virus in the clarified allantoic fluid was assessed by the HA assay method. Briefly, 50 µL of sample were added to 96-well V-bottom plates (Nunc) and serially diluted 1:2. Then, 50 µL of 0.5% v/v turkey or chicken red blood cells (RBCs) in PBS were added to each well, and plates were incubated on ice or at 4°C for 45 min. The HA titer was calculated as the highest dilution showing an RBC tear drop after tilting the plate to 90° for 10–20 s.

### 2.5 ELISA

Immulon 4HBX plates (Thermo Fisher Scientific) were coated with 5 μg/mL of inactivated split virus (50 µL per well) in PBS (pH = 7.4) O/N at 4°C. The next day, plates were washed three times with PBS containing 0.1% v/v Tween 20 (PBS-T) and blocked in blocking solution (3% v/v goat serum, 0.5% w/v non-fat dry milk in PBS-T) for 1 h at RT. After blocking, mAbs were added to the first well at a final concentration of 30 μg/mL in blocking solution (150 µL/well). mAbs were then serially diluted 1:3 (100 µL/well) in blocking solution and incubated for 2 h at 20°C. Afterwards, plates were washed three times with PBS-T before adding sheep anti-mouse peroxidase conjugated IgG (H&L, Rockland) in blocking solution (100 µL/well). Plates were incubated for 1 h at 20°C and then washed four times with PBS-T while shaking. To develop plates, 100 µL of *O*-phenylenediamine dihydrochloride (OPD) substrate (SigmaFast OPD, Sigma-Aldrich) was added to each well. After 10 min incubation, the reaction was stopped by adding 50 µL of 3 M hydrochloric acid (HCl) to each well. The optical density was measured at 490 nm (OD_490_) on a Synergy H1 microplate reader (BioTek). The endpoint titer or minimum binding concentration was defined as the final dilution of the mAb at which the signal was greater than the mean plus three times the standard deviation of blank wells on a given plate using the GraphPad Prism 9 software. Cutoff values were calculated independently for each plate.

### 2.6 Competition ELISA

Immulon 4HBX plates were coated with 5 μg/mL of inactivated split virus (50 µL per well) in PBS (pH = 7.4) O/N at 4°C. Plates were washed three times with PBS-T and blocked in blocking solution for 1.5 h at RT. After blocking, competing mAbs at a concentration of 20 μg/mL (100 µL/well) were added and incubated for 2 h at 20°C. Afterwards, plates were washed three times with PBS-T, and the target biotinylated mAb was added. The biotinylated mAb was added to the first row at a concentration of 30 μg/mL (150 µL/well), serially diluted 1:3 in blocking solution, and incubated for 2 h at 20°C. Plates were washed three times with PBS-T and subsequently incubated with streptavidin conjugated to horseradish peroxidase (Thermo Fisher Scientific) in blocking solution (100 µL/well). After 1 h incubation at 20°C, plates were washed four times with PBS-T with shaking and then developed with 100 µL of OPD substrate per well. After 10 min incubation, the reaction was stopped by adding 50 µL of 3 M HCl to each well. The OD_490_ was measured on a Synergy H1 microplate reader. The data were analyzed using GraphPad Prism 9 and values were expressed as area under the curve (AUC). The cutoff was defined as the average of all blank wells plus three times their standard deviation.

### 2.7 Sandwich ELISA

The sandwich ELISA method was adapted and further optimized from (Rajendran et al., 2018). Immulon 4HBX plates were coated with 2 μg/mL of an anti-HA head mAb (100 µL per well) in PBS (pH = 7.4) O/N at 4°C. The following day, plates were washed three times with PBS-T and were blocked in blocking solution for 1.5 h at RT. In the meantime, purified virus preparations were diluted 1:10 in blocking solution containing 0.05% v/v zwittergent 3–14 detergent (EMD Millipore), and recombinant HA protein standards diluted to 16 μg/mL in blocking solution containing 0.05% v/v zwittergent 3–14 detergent. The dilutions were incubated at RT for 1 h. After blocking, 150 µL of test antigen or recombinant HA protein standard was added to the first well and then serially diluted 1:3 in blocking solution (100 µL/well). The plates were incubated for 2 h at 20°C and washed three times with PBS-T. After incubation, 100 µL of a biotinylated anti-HA stalk mAb diluted to 5 μg/mL in blocking solution was added per well. Plates were again incubated for 1h at 20 °C and subsequently washed three times with PBS-T. Afterwards, 100 µL of 1:3,000 diluted streptavidin conjugated to horseradish peroxidase was added per well and incubated for 1h at 20°C. Then, plates were washed four times with PBS-T while shaking and developed with 100 µL of OPD substrate per well. After developing for 10 min, the reaction was stopped by addition of 3 M HCl and read at an absorbance of 490 nm on a Synergy H1 microplate reader. The 50% effective concentration (EC_50_) values were calculated, and the absolute HA concentrations in the test samples were obtained with relation to the recombinant HA protein standards by parallel line analysis/EC50 comparison using GraphPad Prism 9 (Rajendran et al., 2018).

### 2.8 NA-Star assay

The enzymatic activity of the NA from cHA and mHA viruses was determined by using the NA-Star™ Influenza NA Inhibitor Resistance Detection kit (Applied Biosystems) according to manufacturer’s instructions. A total protein concentration in the range of 5–50 μg/mL was used as the starting dilution to measure the NA activity of live, inactivated, and inactivated split viruses. Samples were serially diluted 1:2 across the plate. The read-out was based on the luminescence signal measured in a Synergy H1 hybrid multimode microplate reader. The NA activity was measured as the AUC using GraphPad Prism 9.

### 2.9 Measurement of Triton X-100 content

Residual Triton X-100 content in inactivated split virus samples was measured by absorbance at 280 nm. Test samples were mixed 1:1 with pure methanol and centrifuged at 25,000 ×g for 30 min at RT. A standard curve of known concentrations of Triton X-100 in pure methanol was also included for absolute Triton X-100 quantification. 80–90 μL of the supernatant was transferred to 96-well UV plates (Corning), and the absorbance at 280 nm was measured in a Synergy H1 hybrid multimode microplate reader. The amount of Triton X-100 in test samples was calculated using Excel (Microsoft) according to the Triton X-100 standard curve and plotted with GraphPad Prism 9.

### 2.10 Quantification of ovalbumin

The quantity of ovalbumin in live, inactivated, and inactivated split virus samples was measured by ELISA according to the manufacturer’s instructions (Morinaga). Briefly, 40 μL of sample was mixed in 760 μL of sample extraction solution (sample buffer, extraction component A, 2-mercaptoethanol, distilled water at a ratio 5:5:2:88, respectively), vortexed for 30 s, and heated at 90°C–95°C for 10 min. Then, samples were vortexed for 30 s, and centrifuged at 3,000 ×g at RT for 20 min. The supernatant was diluted depending on the sample in the range of 1:1,000 to 1:10^6^ in diluent I (sample buffer and distilled water at a ratio of 1:20). An ovalbumin protein standard (egg standard) was also included for absolute ovalbumin protein quantification. 100 μL of sample or standard were dispensed per well of the antibody-coated microplate module and incubated at 20°C for 1 h. Plates were washed six times with washing solution (wash buffer and distilled water at a ratio of 1:20), and 100 μL of enzyme-conjugated antibody was added per well and incubated at 20°C for 30 min. Afterwards, plates were washed six times with washing solution, and 100 μL of enzyme substrate was added per well and incubated at 20°C for 30 min. The colorimetric reaction was stopped by the addition of 100 μL of stop solution. Plate read-out was measured at an absorbance of 450 nm on a Synergy H1 hybrid multimode microplate reader. The amount of ovalbumin in test samples was calculated in Excel according to the ovalbumin standard curve and plotted with GraphPad Prism 9.

### 2.11 Sodium dodecyl-sulfate polyacrylamide gel electrophoresis (SDS-PAGE)

SDS-PAGE was performed to characterize live, inactivated, and inactivated split virus samples. Before running the SDS-PAGE, samples were deglycosylated with rapid peptide:N-glycosidase (PNGase) F (New England Biolabs) according to the manufacturer’s instructions for a better resolution of the bands. After deglycosylation, 20 μL of sample was mixed with 2X Laemmli buffer (BioRad) containing 50 mM NuPAGE sample reducing agent (dithiothreitol, Thermo Fisher Scientific). Samples were then incubated for 10–15 min at 90°C–95°C and run on a 4%–20% precast polyacrylamide Mini-PROTEAN TGX gel (BioRad) for 1 h at 120 V (30 μL/well). The gel was stained with SimplyBlue SafeStain (Novex) O/N, and destained in distilled water. Images were taken in a Chemidoc MP Imaging System using the Image Lab software (BioRad).

### 2.12 Dynamic light scattering

The average size of live, inactivated, and inactivated split viruses was assessed in a Litesizer 500 dynamic light scattering (DLS) instrument (Anton Paar) at an angle of 175° with 10 × 12 × 45 mm polystyrene cuvettes (Sarstedt) and 1 mL of sample. Triplicate measurements were performed per sample, each with 11 runs, and processed with the Kalliope software (Anton Paar). Live and inactivated virus samples were diluted 1:1,000 in 0.22 µm filtered PBS (pH = 7.4), while inactivated split virus samples were diluted 1:100.

### 2.13 Transmission electron microscopy

Samples for transmission electron microscopy (TEM) were prepared by the negative staining method using a 2% w/v solution of uranyl acetate (Electron Microscopy Sciences) in distilled water. Virus samples were diluted to approximately 20–100 μg/mL protein in Tris-buffered saline (10 mM Tris, 150 mM NaCl, pH = 7.5). Briefly, 3 μL of diluted virus sample was applied to glow discharged (PELCO easiGlow, TED PELLA) formvar/copper support film TEM grids (Electron Microscopy Sciences). Following a brief incubation of 30 s, the sample was blotted away from the grid using filter paper (Whatman). Sample-laden grids were washed twice by contact with two successive droplets of distilled water, and subsequently dabbed twice into droplets of uranyl acetate solution. Micrographs were obtained using a Hitachi 7,500 (Hitachi) TEM at the Icahn School of Medicine at Mount Sinai, and a Tecnai F20 (Field Electron and Ion company) and JEOL JEM-1230 (Gatan) TEMs at the New York Structural Biology Center.

## 3 Results

### 3.1 Screening of monoclonal antibodies and quantification of prefusion HA content

**FIGURE 1:**
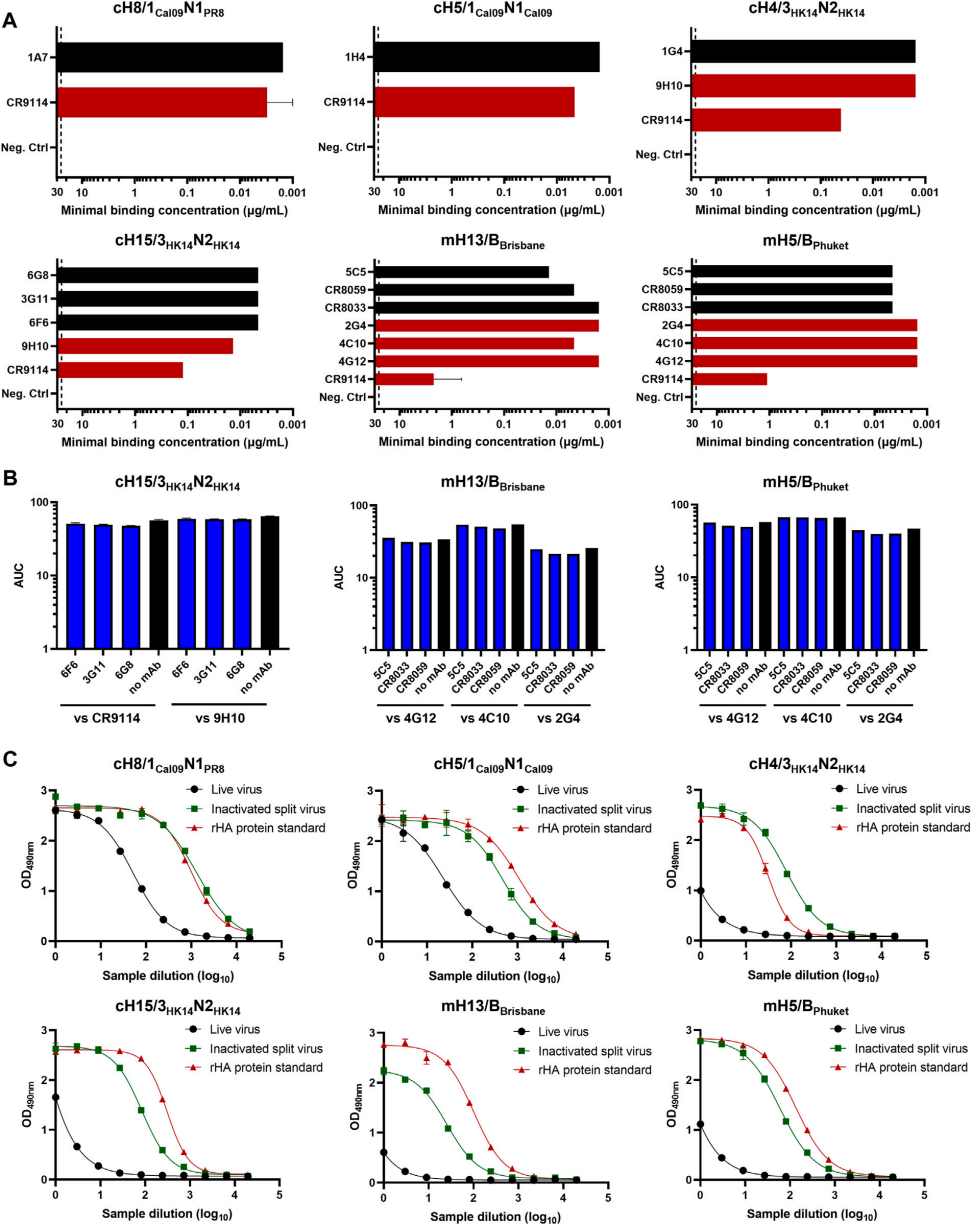
Evaluation of different mAbs for sandwich ELISA against cHA and mHA virus samples. **(A)** The binding of different mAbs targeting the HA head (black) or stalk domain (red) of inactivated split viruses was tested by indirect ELISA. The dashed line indicates the limit of detection (LoD) of the assay. mAbs at concentrations equal or higher than 25 μg/mL showing no binding were considered as no binders. **(B)** Assessment of competition between anti-HA head and stalk mAbs by competition ELISA. Black bars indicate the binding of anti-stalk mAbs in the absence of anti-head mAbs. **(C)** Quantification of prefusion HA in inactivated split virus samples using the mAb combination for each cHA virus: cH8/1_Cal09_N1_PR8_ (1A7 and CR9114), cH5/1_Cal09_N1_Cal09_ (1H4 and CR9114), cH15/3_HK14_N2_HK14_ (3G11 and CR9114), cH4/3_HK14_N2_HK14_ (1G4 and CR9114), mH13/B_Brisbane_ (CR8033 and 4C10), mH5/B_Phuket_ (CR8033 and 4C10) virus. ELISAs were conducted in duplicate, the average and standard deviation between duplicate measurement is shown.

Different monoclonal antibodies (mAbs) targeting the HA head or stalk domains of diverse cHA and mHA constructs were initially screened as candidates for prefusion HA quantification by ELISA. Some of these mAbs had already been previously characterized (Rajendran et al., 2018). The basis of this assay is the binding of an anti-HA head and a stalk mAb to test samples containing HA. If the HA stalk is in the prefusion conformation, both mAbs will be able to bind and a signal will be measured since the anti-HA stalk antibodies are conformation dependent. Of note, while we wanted to select anti-stalk mAbs that would only bind to the prefusion conformation, the anti-head antibodies were mostly chosen based on binding independently of conformation. In fact, most anti-head antibodies used do bind to linear epitopes since the emphasis is on the stalk, and the head conformation was deemed irrelevant for this vaccine approach. To this end, inactivated split virus samples for our six cHA and mHA virus candidates were produced by inactivation with 0.05% v/v βPL at 4°C for 30 min and splitting with 1% v/v Triton X-100 at room temperature (RT) for 1 h. The mAb pair to quantify prefusion HA in group 1 cHA constructs (cH8/1_Cal09_N1_PR8_ and cH5/1_Cal09_N1_Cal09_) was previously determined (Rajendran et al., 2018), but it is shown here that these mAbs also bind inactivated split viruses (Figure 1A). The same was observed for the different mAbs selected for binding against group 2 cHA (cH15/3_HK14_N2_HK14_ and cH4/3_HK14_N2_HK14_) and mHA influenza B inactivated split viruses (mH13/B_Brisbane_ and mH5/B_Phuket_). No competition for binding to inactivated split viruses was measured in any of the combinations of anti-HA head and stalk mAbs tested, indicating different possibilities of mAb pairs for the sandwich ELISA (Figure 1B). For prefusion HA quantification by sandwich ELISA, anti-HA stalk mAbs were biotinylated to increase the sensitivity of the assay. The mAb pairs previously defined for cH8/1_Cal09_N1_PR8_ (1A7 and CR9114) and cH5/1_Cal09_N1_Cal09_ (1H4 and CR9114) viruses captured prefusion HA in inactivated split virus samples as compared with the recombinant HA (rHA) protein standards used in the assay (Figure 1C). Indeed, these mAb pairs enabled the quantification of HA in group 1 cHA viruses even in allantoic fluid, an interesting point since the concentration of influenza viruses is generally low after harvest for ELISA quantification. For the cH15/3_HK14_N2_HK14_ virus, the combination of 3G11 and CR9114 mAbs proved to be the best choice since the anti-HA stalk mAb 9H10, despite showing a higher binding to the inactivated virus, resulted in lower binding in the sandwich ELISA (Figure 1C and Supplementary Figure S1A). A similar pattern was observed for the cH4/3_HK14_N2_HK14_ virus, where the 1G4 and CR9114 mAb pair provided the best binding results (Figure 1C and Supplementary Figure S1B). As for mH13/B_Brisbane_ and mH5/B_Phuket_ influenza B viruses, the mAb combination providing the best sensitivity results in the sandwich ELISA was the broadly cross-reactive CR8033 and 4C10 mAbs in both cases. One caveat here was, that mAb 4C10 also binds the postfusion conformation (see Discussion).

### 3.2 Impact of virus inactivation and splitting conditions on HA conformation and NA activity

**FIGURE 2:**
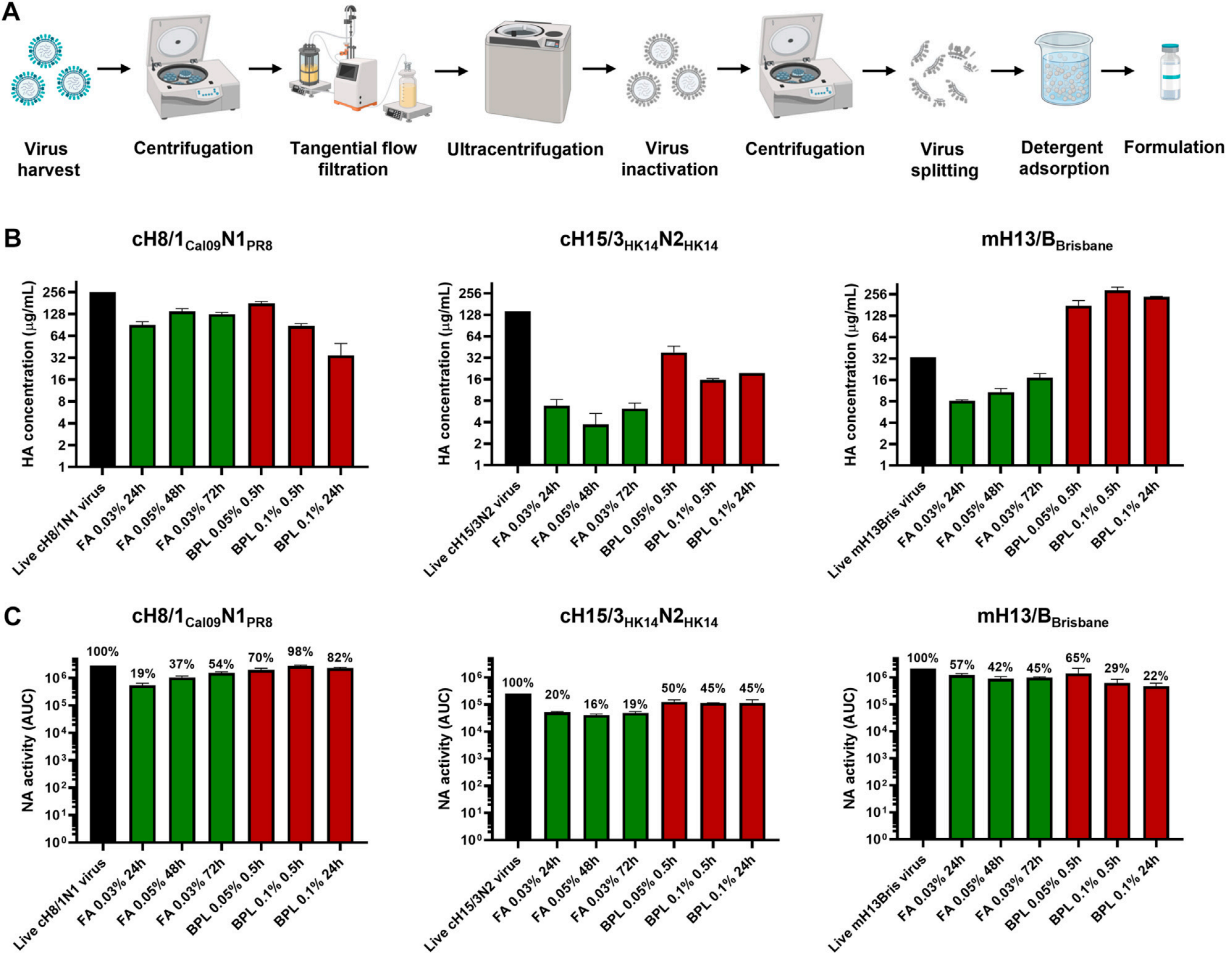
Bioprocess development and inactivation conditions tested in cHA and mHA viruses. **(A)** Schematic of the bioprocess followed for inactivated split vaccine production. Created with BioRender.com. **(B)** Impact of different virus inactivation conditions with formaldehyde (FA, green) and beta-propiolactone (βPL, red) on HA conformation by sandwich ELISA. **(C)** Effect of different virus inactivation conditions with FA (red) and βPL (green) on NA activity using the NA-Star assay. ELISAs and NA-Star assays were conducted in duplicate, the average and standard deviation between duplicate measurement is shown.

**FIGURE 3:**
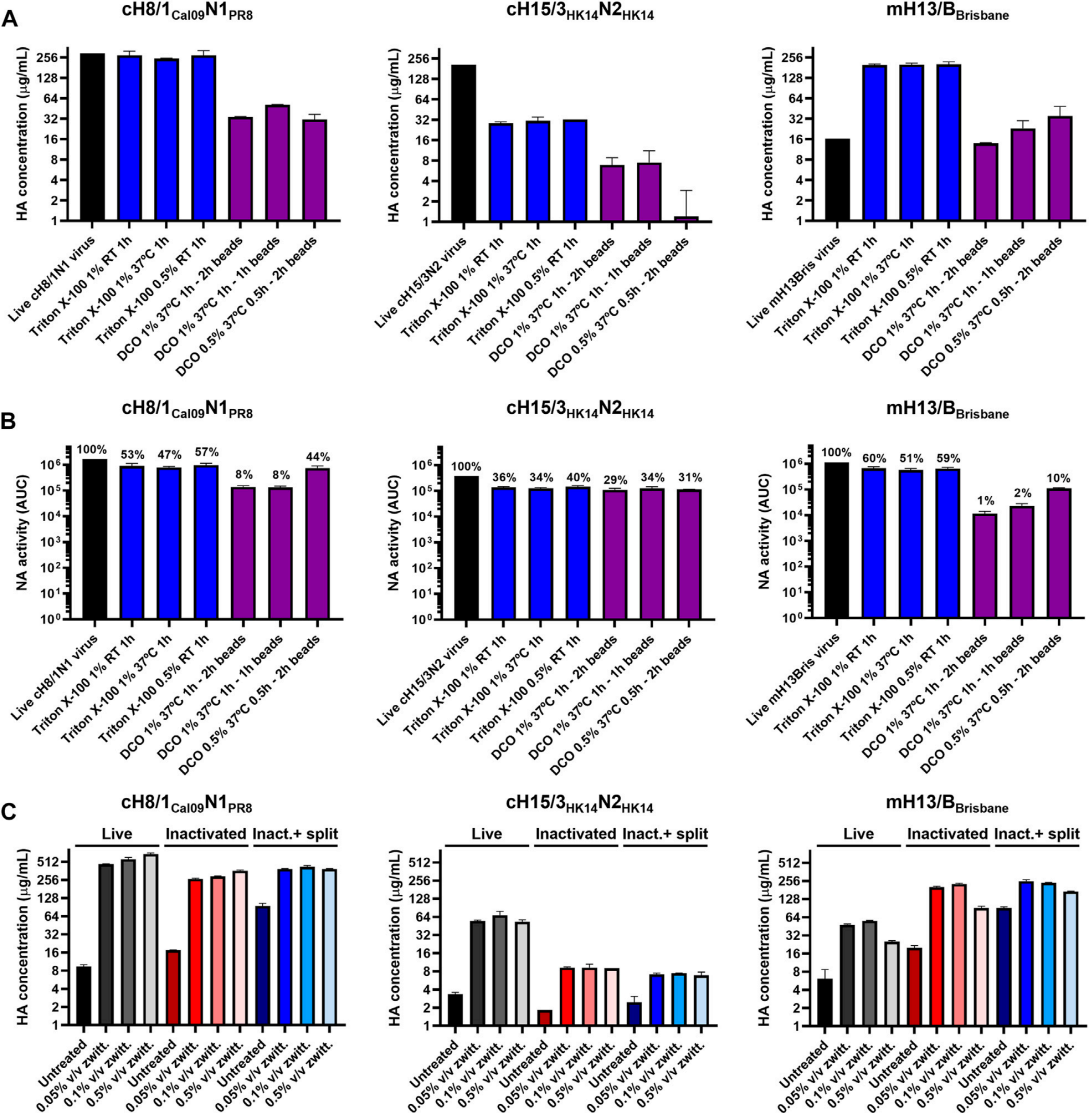
Assessment of different virus splitting conditions and increased amounts of zwittergent 3–14 detergent. **(A)** Impact of different virus splitting conditions with Triton X-100 (blue) and sodium deoxycholate (DCO, purple) on HA conformation after detergent removal by sandwich ELISA. **(B)** Effect of different virus splitting conditions with Triton X-100 (blue) and DCO (purple) on NA activity after detergent removal using the NA-Star assay. **(C)** Measurement of prefusion HA concentration in live, inactivated, and inactivated split virus samples (after Triton X-100 removal with bio-beads) after incubation with different concentrations of zwittergent 3–14 detergent in a sandwich ELISA. Inact: inactivated. ELISAs and NA-Star assays were conducted in duplicate, the average and standard deviation between duplicate measurement is shown.

One influenza virus from each group, cH8/1_Cal09_N1_PR8_, cH15/3_HK14_N2_HK14_, and mH13/B_Brisbane_ respectively, were selected to evaluate the impact of different methods of virus inactivation and splitting with the aim to increase the amount of prefusion HA and neuraminidase (NA) activity in the final vaccine preparations. The main steps of the bioprocess followed are summarized in Figure 2A. A virus concentration step by tangential flow filtration (TFF) was successfully incorporated in the bioprocess toward reducing the volume and therefore the number of ultracentrifugation (UC) rounds that followed. Proportional HA titers before and after the TFF step (10-fold volume reduction) could be measured, without detectable virus loss in the filtrate (Supplementary Figure S2). After UC, different virus inactivation conditions were screened (Tables 1, 2), with βPL yielding higher levels of prefusion HA for the three viruses tested, especially the condition with 0.05% v/v at 4°C for 30 min (Figure 2B). A similar outcome could be observed in terms of retained NA activity (Figure 2C). All inactivation conditions tested with βPL and FA resulted in complete virus inactivation since negative HA titers were obtained after egg re-injection with inactivated viruses. To explore the different virus splitting conditions, a new batch of inactivated virus was produced in eggs using 0.05% v/v of βPL at 4°C for 30 min. From the various splitting protocols tested, all conditions with Triton X-100 had a milder impact on HA conformation and NA activity than splitting with DCO, without significant differences between the Triton X-100 conditions tested (Figures 3A, B).

**TABLE 1:** Different conditions employed for cHA and mHA influenza virus inactivation.

Inactivating agent	Concentration (% v/v)	Time (h)	Temperature (°C)
Formaldehyde (FA)	0.03	24	RT
	0.03	72	4
	0.05	48	4
β-propiolactone (βPL)	0.05	0.5	4
	0.1	0.5	4
	0.1	24	RT

**TABLE 2:** Different conditions employed for cHA and mHA influenza virus splitting.

Splitting agent	Concentration (% v/v)	Time (h)	Temperature (°C)	Time with beads (h)	Temperature with beads (°C)
Triton X-100 (TX-100)	1	1	RT	O/N	4
	1	0.5	37	O/N	4
	0.5	1	37	O/N	4
Sodium deoxycholate (DCO)	1	1	37	O/N	37 (2 h) - 4 (O/N)
	1	1	37	O/N	37 (1 h) - 4 (O/N)
	0.5	1	37	O/N	37 (2 h) - 4 (O/N)

An increase in the concentration of prefusion HA was observed after inactivation and splitting for some of the viruses (Figures 2B, 3A), suggesting better access to the epitopes of the selected mAbs and a better comparability with the recombinant protein standard. Increasing the standard concentration of zwittergent 3–14 detergent used in live, inactivated, and inactivated split virus samples in the sandwich ELISA from 0.05% to 0.5% v/v did not improve the solubility of membrane bound HA (Figure 3C). This indicated that all the HA was already solubilized with the standard sandwich ELISA conditions used.

### 3.3 Assessment of Triton X-100 and ovalbumin content in inactivated split vaccines

**FIGURE 4:**
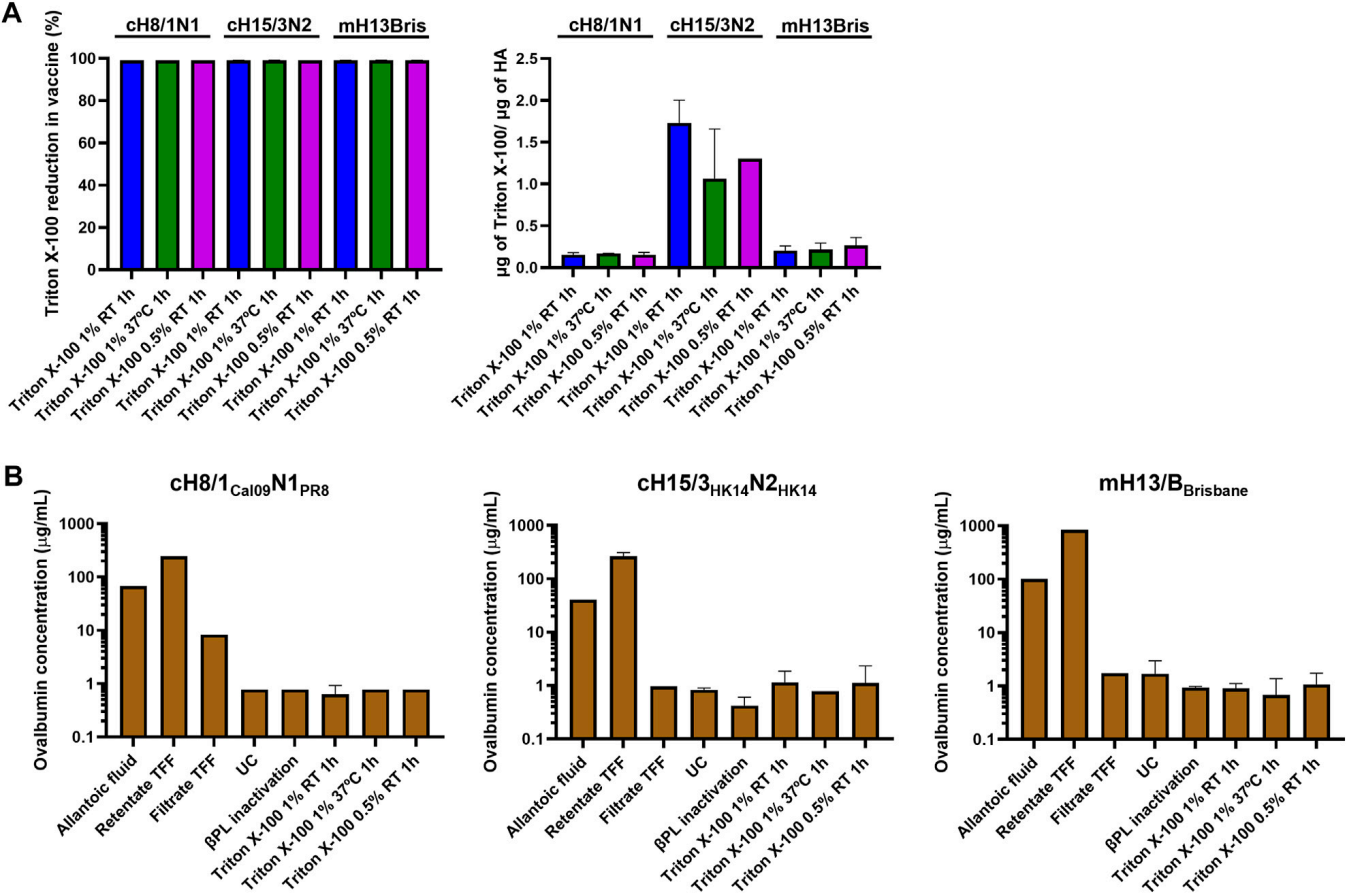
Measurement of residual Triton X-100 and ovalbumin content. **(A)** Quantification of the ratio of residual Triton X-100 per prefusion HA, and reduction of Triton X-100 in different conditions of inactivated virus splitting after Triton X-100 removal with bio-beads. **(B)** Measurement of ovalbumin concentration from harvest of allantoic fluid to the inactivated split virus after Triton X-100 removal with bio-beads. UC: ultracentrifugation, TFF: tangential flow filtration.

Triton X-100 was selected as the detergent for splitting over DCO since it proved to be the best option to retain HA conformation and NA activity. The amount of residual Triton X-100 in the three splitting conditions tested for each virus after detergent removal with hydrophobic beads was below the 5% of the initial amount of Triton X-100 used for splitting. The ratio of residual Triton X-100 to HA content was also below 2 μg/μg HA (Figure 4A). The content of ovalbumin (OVA), the main protein contaminant from the eggs, was below 2 μg/mL in all three Triton X-100 splitting conditions (Figure 4B). Interestingly, the virus concentration step by TFF filtration was not very effective at removing the OVA from the virus samples. The UC step proved to be key in reducing the amount of OVA protein to very low levels in the final vaccine preparation.

### 3.4 Characterization of the effect of inactivation and splitting on cHA and mHA influenza viruses

**FIGURE 5:**
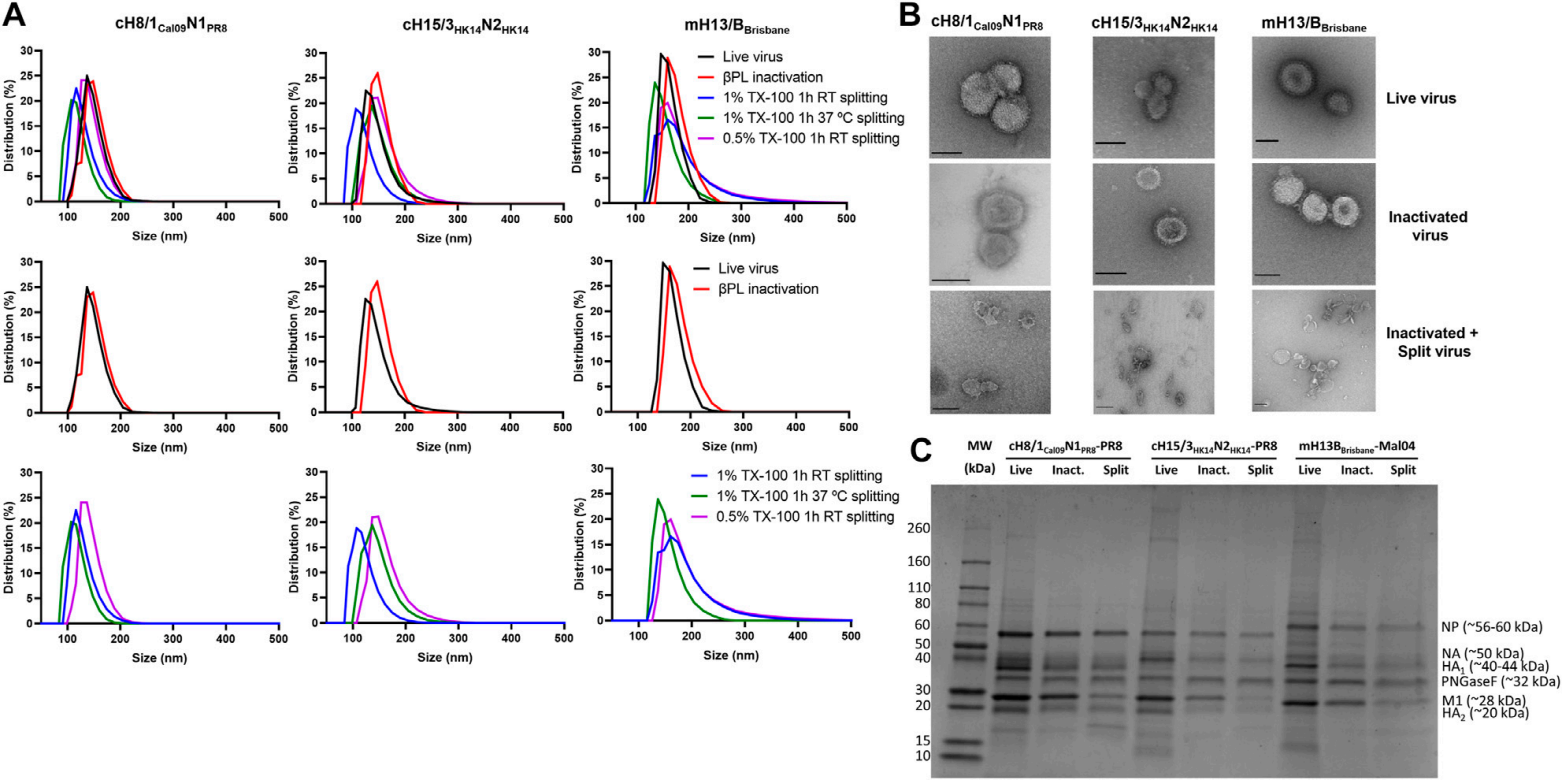
Characterization of live, inactivated, and inactivated split virus samples after Triton X-100 removal with bio-beads. **(A)** Measurement of the size of live, inactivated, and split cHA and mHA viruses by dynamic light scattering. **(B)** Transmission electron microscopy images of live, inactivated, and inactivated split virus samples. Scale bar: 100 nm. **(C)** SDS-PAGE separation of proteins on a 4%–20% polyacrylamide gel stained with SimplyBlue SafeStain. The putative positions of the viral proteins, identified by their predicted molecular weights, are indicated. TX-100: Triton X-100.

The impact of inactivation and splitting on cHA and mHA virus size was analyzed by dynamic light scattering. No remarkable impact on virus size was observed after inactivation with 0.05% v/v βPL (Figure 5A), with virus particle sizes in the range of 100–200 nm for cH8/1_Cal09_N1_PR8_ and cH15/3_HK14_N2_HK14_ viruses, and from 130–230 nm for the mH13/B_Brisbane_ virus. Virus splitting with 0.5% v/v Triton X-100 resulted in a less decrease in particle size in comparison with 1% v/v Triton X-100 splitting, possibly indicating a lower level of virus disruption achieved in the former. Virus splitting with 1% v/v Triton X-100 at RT or 37°C yielded similar levels of virus disruption. Therefore, splitting with 1% v/v Triton X-100 at RT for 1h was the condition selected for the final bioprocess. Characterization of the three cHA and mHA influenza viruses after UC (live virus), after 0.05% v/v βPL inactivation (inactivated virus), and after 1% v/v Triton X-100 splitting and detergent removal with bio-beads (inactivated split virus) was performed by TEM (Figure 5B). No morphological and size changes were detected between live and inactivated virus samples, while virus splitting resulted in heterogenous species, ranging from disc-like particles to smaller structures. Analysis of these different samples for the three viruses in a reducing SDS-PAGE gel after deglycosylation showed the major viral proteins of the influenza virus, including cleaved HA_1_ and HA_2,_ the nucleoprotein (NP), the matrix protein 1 (M1), and some faint bands that might represent the NA and the polymerase proteins (Figure 5C). A general decrease in the presence of all virus proteins was observed after inactivation and especially after splitting, in line with the reduction of HA and NA content previously shown (Figures 2B, C, 3A, B). Importantly, there was no obvious contamination with egg-derived material.

## 4 Discussion

The development of a universal influenza virus vaccine that provides protection against any influenza virus is one of the most critical public health priorities (Krammer et al., 2018a). Among the current strategies to develop a universal influenza virus vaccine, sequential vaccination with cHA constructs that refocus the immune response to the immunosubdominant but also more conserved stalk domain of the HA holds great promise (Nachbagauer et al., 2021). In this work, we aimed at developing a bioprocess that allows the production of inactivated split cHA and mHA vaccine for pre-clinical testing in different animal models, and set the basis for the bioprocess to manufacture inactivated split vaccine for clinical trials in humans in the near future.

One important aspect to consider with our cHA and mHA constructs is that the HA stalk should be in the prefusion conformation. The single radial immunodiffusion assay, which is considered as the gold standard to measure HA concentration, is not able to distinguish between HAs with a native or denatured stalk domain (Schild et al., 1975). To this end, a methodology based on a sandwich ELISA was previously developed to quantify prefusion HA. This technique was successfully validated with our group 1 cHA virus candidates (cH8/1_Cal09_N1_PR8_, cH5/1_Cal09_N1_Cal09_) after virus inactivation (Rajendran et al., 2018). In this work, we showed that the mAb pairs selected for these viruses, 1A7/CR9114, and 1H4/CR9114, respectively, are also useful to quantify prefusion HA in inactivated split virus samples. The mAb pairs for group 2 cHA viruses cH15/3_HK14_N2_HK14_ (3G11/CR9114) and cH4/3_HK14_N2_HK14_ (1G4/CR9114), and influenza B mHA viruses mH13/B_Brisbane_ and mH5/B_Phuket_ (CR8033/4C10) were also determined, proving that these mAb pairs can be used to quantify prefusion HA in live, inactivated, and inactivated split virus samples by sandwich ELISA. However, changes in binding of the mAb pairs to these different forms of the virus were detected, especially for the mH13/B_Brisbane_ virus. We hypothesized that the accessibility of the mAb pair to their HA epitopes in the inactivated and inactivated split samples might be enhanced after the inactivation and splitting step. However, increasing the quantity of zwittergent 3–14 detergent, a mild ionic agent used in the sandwich ELISA for membrane permeabilization and disruption of aggregated HA molecules, did not further improve the binding of these mAbs. Changes in HA structure that alter the binding to the epitopes targeted by these mAbs might explain the increase in HA concentration after inactivation and splitting. In contrast to the influenza A virus HA stalk mAbs used, which are selective for the prefusion conformation of the HA, mAb 4C10 also binds well to the postfusion conformation. Therefore, the sandwich ELISA might be further improved for influenza B mHA viruses in the future with mAbs targeting prefusion-only conformational epitopes in the stalk domain. Additionally, we envision the use of the sandwich ELISA to measure HA conformation in stability studies in combination with reversed-phase high-performance liquid chromatography for a more accurate HA quantification (Kapteyn et al., 2006).

Inactivated split influenza virus vaccine production is mainly conducted in embryonated chicken eggs (Krietsch Boerner, 2020), though efforts are being devoted to transitioning toward cell-based (Imran et al., 2022) or recombinant HA vaccines (Richards et al., 2020). We aimed at developing a bioprocess for our cHA and mHA-based vaccines that can use the existing egg-based manufacturing capacity. A TFF step was successfully implemented between the clarification and UC steps with little to no virus loss in order to reduce the number of UC rounds required to process the clarified allantoic fluid. The impact of different virus inactivation conditions with FA and βPL on prefusion HA, but also on the amount of enzymatically active NA, were assessed. The NA, despite neither being quantified nor standardized in commercial influenza vaccines, also plays a role in protection and reduction of viral shedding (Monto et al., 2015; Krammer et al., 2018b). Inactivation with 0.05% v/v βPL at 4°C for 30 min generally retained a higher level of prefusion HA and active NA. This is in line with previous studies showing higher HA recoveries after inactivation with βPL compared with FA (Budimir et al., 2012; Kon et al., 2016). Furthermore, inactivation with βPL is regarded as safer since it can be inactivated with temperature, while additional steps such as diafiltration need to be considered for FA removal. However, the outcome of inactivation is also dependent on other factors such as reagent or virus concentration, buffers, virus strain, incubation time, and temperature since inactivation with FA has also been reported to have a lower impact on antigen integrity (Bonnafous et al., 2014; Herrera-Rodriguez et al., 2019). Different virus splitting conditions with Triton X-100 and DCO were also evaluated. In all cases, splitting with Triton X-100 yielded the best results in terms of prefusion HA and active NA. These differences might be attributed to the pre-incubation step at 37°C in DCO split viruses to avoid sample solidification. Among the different conditions with Triton X-100 tested, splitting with 1% v/v Triton X-100 was the one selected since it provided a higher degree of virus disruption. Whereas no changes in morphology or structure were appreciable after virus inactivation, virus splitting with Triton X-100 resulted in a variety of specimens including small particles that could be related to HA complexes, and disc-like structures that could represent micelles or not fully disrupted viruses. A similar observation has recently been described in the characterization of different commercial influenza virus vaccines (Myers, 2022).

The presence of the specific contaminants OVA and Triton X-100 was also addressed. No specific guidelines on the amount of residual Triton X-100 in the final influenza virus vaccine preparation are available, but we wanted to keep the detergent to a low level (Herman et al., 2021) while not fully removing it since it has been reported that the presence of Triton X-100 helps to improve the stability and reduce the aggregation of HA (Rhodes et al., 2015). To this end, samples were incubated with hydrophobic beads (Holloway, 1973) and the amount of Triton X-100 in our final vaccine preparations was reduced below 0.05% v/v (<2 μg/μg HA), which is in agreement with the maximum ratio of residual Triton X-100/HA reported in the information leaflet of commercial influenza vaccines. The OVA from eggs is another contaminant of this platform that has to be kept at low levels, especially considering that some people might develop a severe reaction to this egg component (Gruenberg and Shaker, 2011). The UC step was proven to be crucial to remove most of the OVA in our final vaccine preparations, attaining a final concentration below 2 μg/mL. This is in the range of maximum residual OVA content in egg-based commercial influenza vaccines (Waibel and Gomez, 2010). Interestingly, the 100 kDa TFF step was only able to remove a fraction of the OVA (∼45 kDa) in the allantoic fluid, possibly indicating that OVA forms aggregates to a certain degree.

In summary, a bioprocess to produce inactivated split cHA and mHA virus vaccines in eggs has been developed. A method based on a sandwich ELISA for prefusion HA quantification in cHA and mHA viruses has also been defined. Virus inactivation with βPL and splitting with Triton X-100 provides the highest yields of prefusion HA and active NA. In addition, the amount of residual Triton X-100 and OVA is reduced to low levels. This study provides the basis for a bioprocess to produce a cHA and mHA trivalent universal influenza virus vaccine for testing in future clinical trials in humans.

## 5 Sequences

HA gene sequences have been submitted to Genbank and will become available under the following identifiers:

cH5/1_Cal09_N1_Cal09_: OQ349657;
cH8/1_Cal09_N1_PR8_: OQ349625;
cH4/3_HK14_N2_HK14_: OQ349617;
cH15/3_HK14_N2_HK14_: OQ349633;
mH13/B_Brisbane_: OQ349641;
mH5/B_Phuket_: OQ349649.

## Data Availability

The raw data supporting the conclusion of this article will be made available by the authors, without undue reservation.
