# Supplementary material for: Bioprocess development for universal influenza vaccines based on inactivated split chimeric and mosaic hemagglutinin viruses
Source: Front Bioeng Biotechnol. 2023 Jun 5;11:1097349. doi: 10.3389/fbioe.2023.1097349 (PMC10277804; doi:10.3389/fbioe.2023.1097349)
Supplement: Supplementary file 1 [file DataSheet1.docx]

Bioprocess development of universal influenza vaccines based on inactivated split chimeric and mosaic hemagglutinin viruses

Eduard Puente-Massaguer ^1^, Annika Beyer ^1^, Madhumathi Loganathan ^1^, Iden Sapse ^1^, Juan Manuel Carreño ^1,2^, Goran Bajic ^1^, Weina Sun ^1^, Peter Palese ^1,3^, Florian Krammer ^1,2,4,*^

^1^ Department of Microbiology, Icahn School of Medicine at Mount Sinai, New York, NY 10029, USA.

^2^ Center for Vaccine Research and Pandemic Preparedness (C-VaRPP), Icahn School of Medicine at Mount Sinai, New York, NY 10029, USA.

^3^ Department of Medicine, Icahn School of Medicine at Mount Sinai, New York, NY 10029, USA.

^4^ Department of Pathology, Icahn School of Medicine at Mount Sinai, New York, NY 10029, USA.

***Corresponding author:** Florian Krammer

**E-mail:** [florian.krammer@mssm.edu](mailto:florian.krammer@mssm.edu)

**
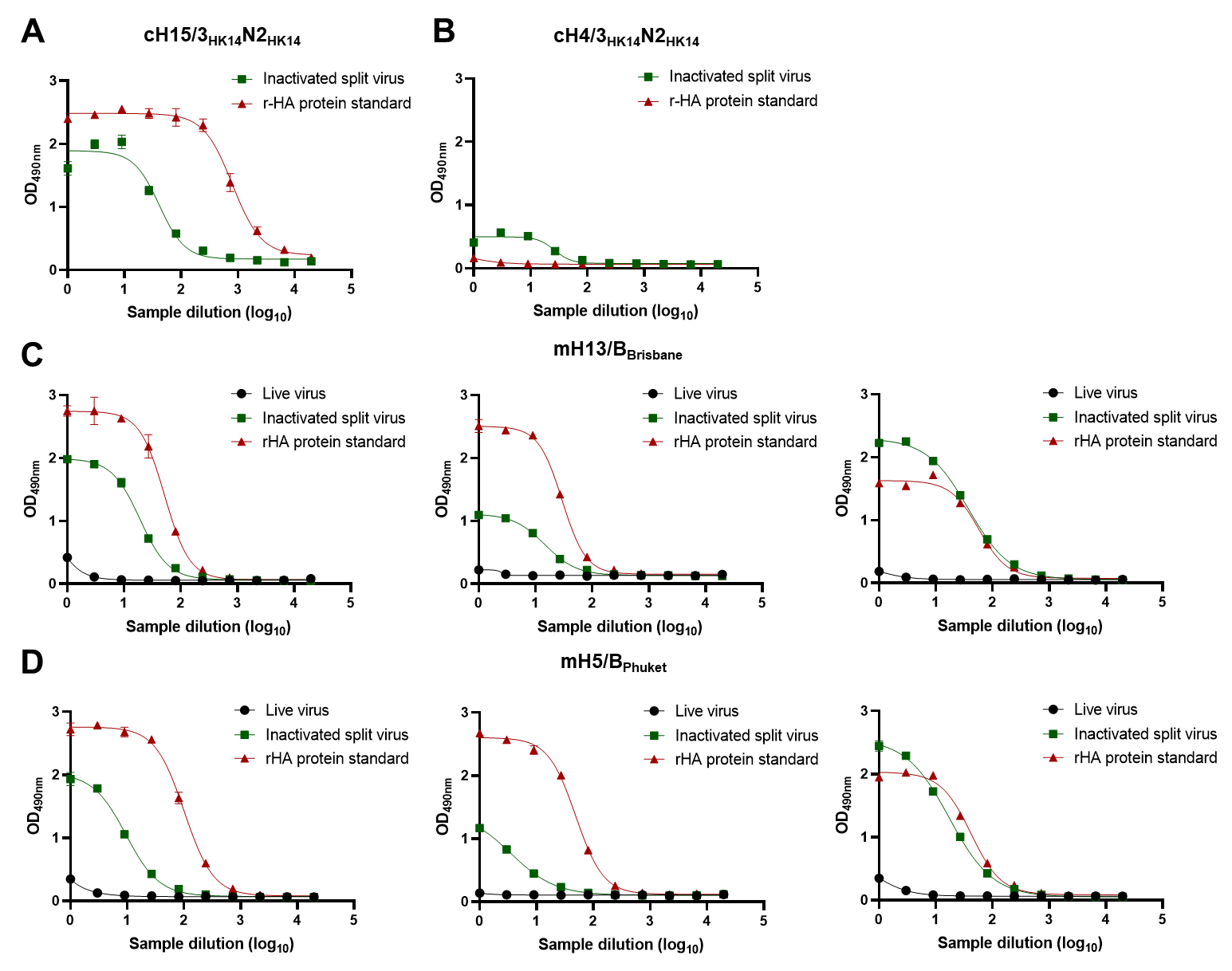
**

**Figure S1**. Evaluation of different mAbs for sandwich ELISA against cHA virus samples. (A) Assessment of 3G11 and 9H10 mAbs for HA quantification in cH15/3_HK14_N2_HK14_ virus by sandwich ELISA. (B) Evaluation of 1G4 and 9H10 mAbs for HA quantification in cH4/3_HK14_N2_HK14_ virus by sandwich ELISA. (C – D) Assessment of CR8033 and CR9114, CR8059 and CR9114, and CR8059 and 4C10 mAbs for HA quantification in mH13/B_Brisbane_ and mH5/B_Phuket_ viruses by sandwich ELISA (left to right).


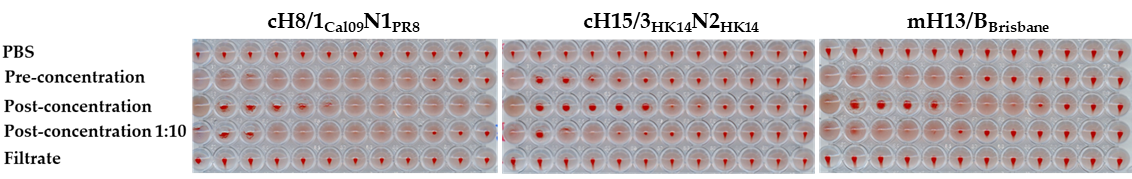


**Figure S2**. Measurement of cHA and mHA virus pre- and post-tangential flow filtration (TFF) step by HA assay. PBS refers to no virus addition, pre-concentration to the amount of virus in the clarified allantoic (before TFF), post concentration to the amount of virus in the retentate (after 10-fold concentration by TFF), post concentration 1:10 to the amount of virus in the retentate 1:10 diluted, and filtrate to the amount of virus in the fluid removed from the system after TFF. 1:2 serial virus dilutions were performed for each sample (from left to right).
